# High Prevalence of Genital Human Papillomavirus Infection in Patients With Primary Immunodeficiencies

**DOI:** 10.3389/fimmu.2021.789345

**Published:** 2021-11-16

**Authors:** Michael Gernert, Matthias Kiesel, Matthias Fröhlich, Regina Renner, Patrick-Pascal Strunz, Jan Portegys, Hans-Peter Tony, Marc Schmalzing, Eva Christina Schwaneck

**Affiliations:** ^1^ Department of Medicine II, Rheumatology and Clinical Immunology, University Hospital of Würzburg, Würzburg, Germany; ^2^ Department of Gynecology and Obstetrics, University Hospital of Würzburg, Würzburg, Germany; ^3^ Institute of Sociology, Friedrich Alexander University of Erlangen, Erlangen, Germany; ^4^ Rheumatology and Clinical Immunology, Asklepios Klinik Altona , Hamburg, Germany

**Keywords:** human papillomavirus, primary immunodeficiency, inborn errors of immunity (IEIs), common variable immunodeficiency (CVID), genital warts

## Abstract

**Background:**

Genital human papillomavirus (HPV)-infections are common in the general population and are responsible for relevant numbers of epithelial malignancies. Much data on the HPV-prevalence is available for secondary immunodeficiencies, especially for patients with human immunodeficiency virus (HIV)-infection. Little is known about the genital HPV-prevalence in patients with primary immunodeficiencies (PIDs).

**Methods:**

We performed a cross-sectional study of patients with PIDs and took genital swabs from male and female patients, which were analyzed with polymerase chain reaction for the presence of HPV-DNA. Clinical and laboratory data was collected to identify risk factors.

**Results:**

28 PID patients were included in this study. 10 of 28 (35.7%) had HPV-DNA in their genital swabs. 6 patients had high-risk HPV-types (21.4%). Most patients had asymptomatic HPV-infections, as genital warts were rare (2 of 28 patients) and HPV-associated malignancy was absent. Differences in the HPV-positivity regarding clinical PID-diagnosis, duration of PID, age, sex, immunosuppression, immunoglobulin replacement, or circumcision in males were not present. HPV-positive PID patients had higher numbers of T cells (CD3^+^), of cytotoxic T cells (CD3^+^/CD8^+^), of transitional B cells (CD19^+^/CD38^++^/CD10^+^/IgD^+^), and of plasmablasts (CD19^+^/CD38^+^/CD27^++^/IgD^-^) compared to HPV-negative.

**Conclusion:**

PID patients exhibit a high rate of genital HPV-infections with a high rate of high-risk HPV-types. Regular screening for symptomatic genital HPV-infection and HPV-associated malignancy in PID patients seems recommendable.

## Background

Human papillomaviruses (HPV) are a group of DNA viruses, exclusively detected in humans. HPV infects basal epithelial cells, is transmitted through smear infection and is the most common sexually transmitted infection (1). To date, there have been 228 types of HPV identified (2). According to the International Agency for research on Cancer (IARC) (3) HPV types can be grouped according to their oncogenic potential, especially for cervical cancer. Low-risk types are type 6 and 11, for example. These are found in genital warts (condylomata acuminata) and usually do not cause malignancies. High-risk types, i.e. carcinogenic types, are the types 16, 18, 31, 33, 35, 39, 45, 51, 52, 56, 58, and 59. The high-risk types cause precancerous lesions like cervical intraepithelial neoplasia (CIN), but also vulvar, vaginal, anal, and penile intraepithelial neoplasia (VIN, VAIN, AIN, and PIN, respectively). Those lesions can progress into the respective invasive cancers with cervical cancer being the fourth most common cancer in women worldwide (4). Additionally, squamous cell carcinomas of the oral cavity, of the oropharynx, and the tonsils can be caused by HPV (3). The third group of HPV-types includes HPV-types, whose dignity is not yet assured. These HPV-types are named probably or possibly carcinogenic (3).

Asymptomatic genital infections with HPV are common (5) and are defined as detection of HPV-DNA without evidence of HPV-lesions (which comprise all of the afore mentioned clinical detectable HPV-alterations). The mechanism of HPV-DNA transformation into HPV-lesions is widely investigated (1) but the clearance of HPV-DNA and HPV-lesions is not fully understood. In immunocompetent individuals HPV-DNA is often only transiently detectable for 1 to 2 years, with longer persistence of high-risk types (6, 7).

To prevent HPV-infections, in the year 2006 the first HPV-vaccination was approved by the European Medicines Agency (EMA). The HPV-vaccination was therefore the first drug to prevent cancer. Two vaccines are available at present: A bivalent vaccine including HPV 16 and 18 (Cervarix^®^) and a 9-valent vaccine including HPV 6, 11, 16, 18, 31, 33, 45, 52, and 58 (Gardasil^®^9). In Germany, the tetravalent Gardasil^®^ vaccine including HPV 6, 11, 16, and 18 is no longer available (8).

In Germany, since 2018 the competent authority for vaccinations (*Ständige Impfkommission*, STIKO) implemented the vaccination of all girls and boys between the ages of 9 to 14 years in its recommendations [(8, 9) on the basis of (10–12)], implicating that the HPV-vaccination was henceforward covered by the compulsory health insurance. In 2020, the German national guideline for prevention of cervical cancer was updated and newly implemented the screening on HPV (13): From the age of 35 years, women now receive HPV-DNA and cytological testing from cervical swabs every 3 years, in contrast to the former yearly cytological swabs without routine HPV-DNA measuring.

The prevalence of genital HPV in the general population in women is reported to be 11.7% worldwide and 14.2% in Europe (14), in European men it is reported to be 12.4% (15). In systemic lupus erythematosus (SLE) a higher prevalence of HPV-infections has been reported (16) and cellular immunodeficiencies e.g. HIV or renal allograft recipients have a higher risk of HPV-lesions (17, 18). These findings indicate that a disturbance of the immune surveillance leads to higher risk of HPV-associated disease. As few data is available regarding HPV-prevalence in patients with primary immunodeficiencies (PIDs), the aim of our study was to evaluate the prevalence of HPV-infections and HPV-associated diseases in patients with PIDs and to identify risk factors by detailed description of their PIDs’ phenotype.

## Patients and Methods

### Data Acquisition and Classification of Primary Immunodeficiencies

Genital swabs and data acquisition were performed between December 2019 and March 2021. PIDs were diagnosed according to the European Society for Immunodeficiencies (ESID) registry working definitions for clinical diagnosis of PID (19) as for most patients a monogenetic mutation was not detected or genetic testing was not performed. Common variable immunodeficiency patients were classified according to the EUROclass trial (20).

### HPV-DNA Detection

For women a colposcopy and a cervical swab were performed and for men a swab of the glans penis including the introitus urethrae was performed, both with FLOQSwabs in UTM-RT transport medium (both Copan, Brescia, Italy). For detection of HPV-DNA a line probe assay was done (INNO-LiPA^®^ HPV Genotyping Extra II, Fujirebio Europe, Ghent, Belgium; allows genotyping of 32 HPV genotypes: 6, 11, 16, 18, 26, 31, 33, 35, 39, 40, 42, 43, 44, 45, 51, 52, 53, 54, 56, 58, 59, 61, 62, 66, 67, 68, 70, 73, 81, 82, 83, 89). Additionally, to not miss high-risk HPV types the cobas^®^ HPV PCR kit (Roche, Basel, Switzerland) was used (allows single genotyping of HPV subtypes 16 and 18 and gives an overall result of further high-risk HPV genotypes including 31, 33, 35, 39, 45, 51, 52, 56, 58, 59, 66, 68).

### Immunophenotyping

Peripheral blood was collected in EDTA tubes and directly processed. Fluorescence-activated cell sorting (FACS) was performed as formerly described (21, 22). Of each antibody 10 µl were used and incubated for 15 minutes at room temperature. Erythrocytes were lysed (with VersaLyse with IOTest3 Fixative Solution, ratio 2:1, both Beckman Coulter, Krefeld, Germany). Two centrifugation steps followed (each at 300 relative centrifugal force for 15 minutes with resuspensation of the pellet in phosphate buffered saline + 1% fecal calf serum). For characterization of T cells and natural killer (NK) cells the following antibodies were used: CD3-FITC, CD4-APC, CD8 EDC, CD14-APC A700, CCR7-PC7, CD45RA-PB, CD45-Krome Orange, CD56/16-APC A750, γδTCR-PC5.5 (each Beckman Coulter, Krefeld, Germany). Lymphocytes were detected by gating sideward scatter *vs* CD45 and monocytes were excluded by CD14-positivity. Within the lymphocyte gate, T cells were defined as CD3^+^ events, T helper cells as CD3^+^/CD4^+^, cytotoxic T cells as CD3^+^/CD8^+^, NK cells as CD3^-^/CD56/16^+^, NKT cells as CD3^+^/CD56/16^+^, γδ T cells as CD3^+^/gammadelta T cell receptor^+^, naïve T cells as CD3^+^/CCR7^+^/CD45RA^+^ and naïve T helper cells as CD3^+^/CD4^+^/CCR7^+^/CD45RA^+^. For B cell characterization the following antibodies were used: CD10-PE, CD19-PC7, CD20-APC A750, CD27-ECD, CD38-PC5.5, CD45-Krome Orange (each Beckman Coulter, Krefeld, Germany), IgD-FITC (BD Biosciences, San Jose, CA), CD21-PB (Exbio, Prague, Czech Republic). B cells were detected within the lymphocyte gate by sideward scatter *vs* CD45 and CD19-positivity. Within the B cell compartment transitional B cells were defined as CD38^++^/CD10^+^/IgD^+^, naïve B cells as CD27^-^/IgD^+^, pre-switched memory B cells as CD27^+^/IgD^+^, post-switched memory B cells as CD27^+^/IgD^-^, double negative (DN) B cells as CD27^-^/IgD^-^, CD21^low^ B cells as CD19^+^/CD21^-^ and plasmablasts as CD38^+^/CD27^++^/IgD^-^. A Navios 3L10c cytometer (Beckman Coulter, Krefeld, Germany) was used. The detailed gating strategy has previously been described (23). Numbers of lymphocyte subsets were calculated by multiplying the percentages obtained in the FACS analysis with the lymphocyte numbers/µl form the differential blood count. The latter was measured in a XN-550 automated hematology analyzer (Sysmex, Kobe, Japan).

### Statistical Analysis

Testing for normal distribution was done with Shapiro-Wilk tests. Normal distribution was mostly absent, so medians with interquartile ranges (IQR) were shown. To detect differences between unpaired groups, Mann-Whitney U tests were used for continuous variables and Fisher’s exact tests for categorical variables. SPSS Statistics v 26.0 (IBM, Armonk, NY) was used. For data collection Excel (Microsoft, Redmond, WA) was used. When two-tailed *p-*values were less than or equal to 0.05, differences were considered significant.

## Results

### Patients’ Characteristics

The patient cohort comprised several PIDs: 18 patients (64.3%) had a common variable immunodeficiency disorder (CVID), 6 patients (21.4%) had a combined immunodeficiency (CID), 3 patients (10.7%) an IgA with IgG subclass deficiency, and 1 patient (3.6%) an isolated IgG subclass deficiency. The median age of the PID cohort was 38.9 years (range 20.5-72.1), 16 patients (57.1%) were female. The median age at first diagnosis of PID was 31.5 years (interquartile range, IQR 24.5-39.0) and the PID duration until study inclusion was 5.0 (1.3-13.0) years. Autoimmune features of PID were seen in 39.3% of the patients, lymphoproliferation in 46.4%, and granulomatous lesions in 35.7%. Bronchiectases were present in 25.0%. 21 patients (75.0%) received immunoglobulin replacement with a median IgG serum level of 792.5 (621.8-999.5) mg/dl. 8 patients (28.6%) took an immunosuppressive medication. Patients’ characteristics and further details are summarized in **Table 1**.

**Table 1:** Characteristics of the study population.

Characteristics	Total population	HPV status	
		HPV-negative	HPV-positive^*^
Female, n (%)	16 female/28 patients (57.1)	9 HPV-negative female/16 female (56.3)	7 HPV-positive female/16 female (43.8)
Male, n (%)	12/28 (42.9)	9/12 (75.0)	3/12 (25.0)
Age, median years (range)	38.9 (20.5-72.1)	40.6 (20.5-72.1)	36.5 (30.1-66.4)
Age at PID diagnosis, median years (IQR)	31.5 (24.5-39.0)	30.0 (21.0-39.0)	32.0 (28.3-40.5)
PID duration, median years (IQR)	5.0 (1.3-13.0)	5.5 (1.8-17.3)	4.0 (1.0-13.0)
CVID, n (%)	18/28 (64.3)	13/18 (72.2)	5/18 (27.7)
CID, n (%)	6/28 (21.4)	3/6 (50.0)	3/6 (50.0)
IgA with IgG subclass deficiency, n (%)	3/28 (10.7)	2/3 (66.7)	1/3 (33.3)
Selective IgG subclass deficiency, n (%)	1/28 (3.6)	0/1 (0.0)	1/1 (100.0)
IgG replacement, n (%)	21/28 (75.0)	14/21 (66.7)	7/21 (33.3)
IgG level^$^, median mg/dl (IQR)	792.5 (621.8-999.5)	885.0 (727.0-1070.0)	754.5 (527.0-863.8)
IgA level^$^, median mg/dl (IQR)	9.5 (0.0-35.8)	9.5 (0.0-31.3)	7.0 (0.0-100.0)
IgM level^$^, median mg/dl (IQR)	26.0 (7.5-94.5)	19.0 (10.3-115.8)	33.0 (6.0-63.3)
Autoimmune disease^†^, n (%)	11/28 (39.3)	6/11(54.5)	5/11 (45.5)
Lymphoproliferation^‡^, n (%)	13/28 (46.4)	9/13 (69.3)	4/13 (30.8)
Granulomatous lesions^#^, n (%)	10/28 (35.7)	6/10 (60.0)	4/10 (40.0)
Bronchiectasis, n (%)	7/28 (25.0)	5/7 (71.4)	2/7 (28.6)
Immunosuppression^§^, n (%)	8/28 (28.6)	5/8 (62.5)	3/8 (37.5)
Circumcision in male, n (%)	3/12 (25.0)	2/3 (66.7)	1/3 (33.3)
HPV vaccination in female, n (%)	2/16(12.5)	1/2 (50.0)	1/2 (50.0)

CID, combined immunodeficiency; CVID, common variable immunodeficiency; IQR, inter quartile range; HPV, human papillomavirus; PID, primary immunodeficiency.

*No significant differences were detected between HPV-negative and HPV positive regarding the respective characteristic.

^$^Reference values: IgG 690-1600 mg/dl, IgA 88-410 mg/dl, IgM 34-210 mg/dl.

^†^Autoimmune manifestations: In 7 patients thrombocytopenia, 4 hemolytic anemia, 3 Hashimoto’s thyroiditis, 2 diabetes mellitus type I, 1 myositis, 1 polyarthritis, 1 perichondritis.

^‡^Lymphoproliferation: 9 splenomegaly, 5 lymphadenopathy.

Granulomatous lesions: 7 lung, 2 intestine, 2 liver, 1 cerebrum, 1 meninges.

^§^Immunosuppressive medication: In HPV negative: Azathioprine + 5mg prednisolone, 2.5mg prednisolone, sulfasalazine, rituximab (2g 6 weeks before blood collection), 5mg prednisolone. In HPV positive: mycophenolate + 5mg prednisolone, rituximab (2g 14 months before blood collection), rituximab (2g 42 months before blood collection).

### High Rate of HPV-Positive Genital Swabs in PID Patients

10 out of 28 of our PID patients (35.7%) were positive for HPV-DNA in their genital swabs. 7 of the HPV-positive were female and 3 were male. These patients were considered as HPV-infected. 8 of these patients had no visible pathologies and were therefore characterized as having an asymptomatic HPV-infection. Only 2 of the HPV-infected patients (20.0%) had macroscopic pathologies and were therefore characterized as having a symptomatic HPV-infection: One male had two penile condylomata acuminata caused by HPV type 33, which were removed by shave excision. Afterwards the HPV swab was negative. One female had excessive vulvar, vaginal, and perianal condylomata acuminata caused by HPV type 6. Several excisions were unsuccessful. Malignancy was excluded by histology.

In the patients’ history, 5 out of 28 patients (17.9%) had genital warts in their past. 2 of them were HPV-negative at the time of our study and 3 were HPV positive. No former HPV-associated malignancy was reported (**Table 2**).

**Table 2:** Prevalence and characteristics of genital HPV-infections.

	HPV-negative	HPV-positive
Total population, n (%)	18/28 (64.3)	10/28 (35.7)
HPV subtypes	na	6, 33, 45, 51, 51, 51 + 66, 52, 53 + 68, 62, 82
HPV high-risk types, n (%)	na	6/10 (60.0)
Current genital warts/visible pathologies, n (%)	0/18 (0.0)	2/10 (20.0)
Anamnestic genital warts, n (%)	2/18 (11.1)	3/10 (30.0)
Anamnestic HPV-associated malignancy	0/18	0/10

HPV, human papilloma virus; na, not applicable.

### Dominance of High-Risk HPV-Subtypes in Genital Swabs of PID Patients

The 10 HPV-positive PID patients had the following HPV-types in their genital swabs: HPV 6, 33, 45, 51, 51, 51 + 66 (i.e. dual infection), 52, 53 + 68 (i.e. dual infection), 62, and 82. Therefore 6 of the PID patients had high-risk HPV types, which means a prevalence of 60.0% (**Table 2**).

### Low Prevalence of HPV Vaccination in PID Patients

None of the male PID patients were vaccinated against HPV. Two female PID patients were vaccinated against HPV (2 of 16 i.e. 12.5% of the female PID patients), one with Cervarix^®^ and one with tetravalent Gardasil^®^. The female vaccinated with Cervarix^®^ was HPV-negative, the female vaccinated with tetravalent Gardasil^®^ had HPV 62 (which is not included in this vaccine). The median age of the female patients was 38.9 years (range 20.5 - 66.4) and of the male patients it was 38.8 years (range 22.9 - 72.1). This means only two of our patients (the former described women) were able to receive the HPV-vaccination according to the German national guideline due to their age.

### Low Prevalence of Pathologic Cervical Cytology in Female PID Patients

15 of our 16 female PID patients have undergone regular screening for cervical dysplasia and therefore received cervical cytology with Papanicolaou staining from their gynecologists. The classification was done according to the *Münchner Nomenklatur III* (24). The majority (14/15 i.e. 93.3%) exhibited PAP I [correlating with ‘negative for intraepithelial lesion or malignancy’ [NILM] in the Bethesda system (25)], one HPV-negative female had PAP II-a (correlating with NILM). Another female received a cervical biopsy due to condylomata acuminata, revealing no dysplasia and no malignancy.

### Comparison of Clinical Characteristics Between HPV-Positive and HPV-Negative PID Patients

When HPV-negative and HPV-positive patients were compared, no significant differences in sex, age, PID duration, type of PID, receiving immunoglobulin replacement, level of immunoglobulin G in the serum, intake of immunosuppression or circumcision in males were detected (data not shown). A logistic regression analysis could not be performed due to the low sample size.

### HPV-Positive PID Patients Show Higher T Cells, Higher Transitional B Cells and Higher Circulating Plasmablasts

HPV-positive PID patients had higher T cell numbers [1776.2 (IQR 846.7-4426.9)/µl] compared to HPV-negative patients [856.8 (549.5-1290.0)/µl; *p* = 0.035]. Both, T helper cells and cytotoxic T cells were numerically higher in HPV-positive compared to HPV-negative, only in cytotoxic T cells the difference was significant [T helper: 1333.3 (502.4-2125.1)/µl *vs* 631.3 (358.0-926.8)/µl, *p* = 0.057; cytotoxic T: 647.4 (430.4-1936.5)/µl *vs* 393.7 (192.6-522.8)/µl, *p* = 0.009]. Within the B cell compartment HPV-positive PID patients compared to HPV-negative had higher CD19^+^/CD38^++^/CD10^+^/IgD^+^ transitional B cells [14.7 (4.2-24.3)/µl *vs* 2.6 (1.1-6.9)/µl, *p* = 0.045] and higher CD19^+^/CD38^+^/CD27^++^/IgD^-^ circulating plasmablasts [1.1 (0.5-2.1)/µl *vs* 0.4 (0.1-0.9)/µl, *p* = 0.035] **Table 3**.

**Table 3:** Lymphocyte subsets of PID patients comparing those with a genital HPV infection *versus* those without a HPV infection.

	Percentages % (IQR)		Absolute numbers/µl (IQR)	
	HPV-negative	HPV-positive	HPV-negative	HPV-positive
Lymphocytes	na	na	1250.0 (890.0-1845.0)	1705.0 (962.5-3995.0)
	% within the lymphocyte gate			
T cells CD3^+^	76.2 (69.7-79.8)	81.7 (71.9-88.5)	856.8 (549.5-1290.9)	1776.2 (846.7-4426.9)*
Cytotoxic T cells CD3^+^/CD8^+^	29.6 (25.9-33.8)	34.0 (28.1-44.0)	393.7 (192.6-522.8)	647.4 (430.4-1936.5)*
T helper cells CD3^+^/CD4^+^	54.6 (45.5-62.5)	49.6 (41.8-59.2)	631.3 (358.0-926.8)	1333.3 (502.4-2125.1)
CD4^+^/CD8^+^ ratio	1.8 (1.5-2.7)	1.6 (1.0-1.9)	na	na
Naïve T cells CD3^+^/CCR7^+^/CD45RA^+^	29.3 (16.3-36.0)	22.1 (6.9-36.0)	295.0 (152.4-572.9)	332.2 (64.1-783.6)
Total B cells CD19^+^	11.2 (8.8-12.8)	9.1 (4.7-12.7)	158.5 (96.6-190.7)	178.5 (46.5-448.4)
	% within the CD3^+^/CD4^+^ compartment			
Naïve T helper cells CD3^+^/CD4^+^/CCR7^+^/CD45RA^+^	24.4 (9.6-37.8)	12.2 (2.9-39.5)	136.6 (47.1-255.1)	129.4 (34.3-263.2)
	% within the CD19^+^ compartment			
Double negative B cells CD27^-^/IgD^-^	3.1 (1.8-6.9)	2.1 (0.5-4.1)	4.2 (2.8-9.4)	7.3 (0.6-15.9)
Post-switched memory B cells CD27^+^/IgD^-^	4.2 (0.8-6.8)	0.5 (0.2-15.0)	3.9 (0.6-10.9)	1.3 (0.1-55.4)
Pre-switched memory B cells CD27^+^/IgD^+^	12.0 (8.6-20.8)	5.0 (2.8-12.9)	17.6 (5.5-40.3)	13.0 (1.3-62.7)
Naïve B cells CD27^-^/IgD^+^	75.4 (68.2-87.9)	80.6 (47.3-92.8)	111.2 (89.1-143.9)	120.6 (27.7-287.9)
Transitional B cells CD38^++^/CD10^+^/IgD^+^	2.2 (0.7-4.6)	8.0 (1.8-21.4)	2.6 (1.1-6.9)	14.7 (4.2-24.3)*
CD21^low^ B cells CD19^+^/CD21^-^	17.8 (6.5-78.9)	11.3 (5.3-62.6)	15.6 (10.0-57.2)	22.1 (10.9-73.4)
Plasmablasts CD38^+^/CD27^++^/IgD^-^	0.3 (0.2-0.6)	0.7 (0.3-1.2)	0.4 (0.1-0.9)	1.1 (0.5-2.1)*

Shown are medians (IQR, interquartile range); n_HPV-negative_ = 18, n_HPV-positive_ = 10; na, not applicable; *significant (p ≤ 0.05) in a Mann-Whitney U test.

## Discussion

We describe a cohort of patients with primary immunodeficiencies, who exhibit a high rate of HPV-positivity in genital swabs. Most of our female patients had normal cervical cytology. Also male patients mostly had no genital warts. This reveals a high asymptomatic HPV-infection rate in PID patients. The detected HPV-types were mainly high-risk types, but HPV-associated malignancies were not present. The HPV-vaccination rate was low.

To our knowledge, this is the first cross-sectional study investigating the genital HPV-prevalence in patients with primary immunodeficiencies. To date, mainly case reports or series were reported (26), showing that GATA binding protein 2 (GATA2) and CXC motif chemokine receptor type 4 (CXCR4) deficiencies are associated with genital warts (27). Patients with immune dysregulation and combined immunodeficiencies with associated syndromic features are reported to have HPV-associated warts more often (28). The HPV-prevalence of women in the general population with normal cervical cytology is accounted to 9.0% in Western Europe (29). In Germany, the prevalence of the high-risk types HPV16/18 in women with normal cervical cytology is 3.2% (5). However, the HPV-prevalence depends on the age. Women under 25 years of age show the highest rates of HPV-positivity with a decline in age. Women over 35 years have a HPV prevalence between 2 to 12% in Europe (30). In the general male population the prevalence of genital HPV-DNA is reported to be 12.4% (15) with persisting HPV-prevalence independent of age (31). Our cohort had a median age of 38.9 years and showed a prevalence of 35.7%, which is more than the reported prevalence in the general population. Therefore, we assume that patients with PIDs exhibit a higher rate of HPV-positivity in genital swabs compared to the general population.

Patients with secondary immunodeficiencies, especially HIV-infected patients are widely investigated in terms of their HPV-prevalence. Women with HIV-infection and normal cervical cytology are reported to have a cervical HPV-infection rate from 20.3% up to 57.5% and men to have a penile HPV-infection rate from 10.0% up to 49.5% (5). The HPV-prevalence in our PID-cohort therefore lies within the range reported for secondary immunodeficiencies.

Our PID patients showed positivity for HPV 6, 33, 45, 51 (three-times), 52, 53, 62, 66, 68, and 82, which means that 60% were high-risk HPV-types. In contrast to our PID population, high-risk HPV-types found in the general population with normal cervical cytology are mainly HPV 16, 18, 31, 51, and 52 (5, 31), whereas in cervical cancer mainly HPV 16 and HPV 18 are reported (5). HPV 16, HPV 18 or HPV 31 were not detectable in our cohort, which is a difference to the reported general population cohorts. Comparable to the general population, patients with secondary immunodeficiencies (HIV-infected women in Germany, with normal cervical cytology) show the highest HPV-DNA loads for HPV 16, 31, and 56 (32).

As patients with PIDs have a higher risk to develop malignancies, compared to the general population (33, 34) it is important to know about a carcinogenic/high-risk HPV-infection. Whereas health care providers caring for HIV patients are very well aware of the high risk of HPV-related diseases in their patients, the risk of PID patients is not as widely known. Our study implies that PID patients are equally at risk for HPV-infection and might benefit from regular screenings. Structured and intensified follow-ups should be offered to male and female PID patients, if high-risk HPV-DNA is present in their genital swabs.

All of our female patients were initially evaluated for macroscopic cervical pathologies by a gynecologist. These were mostly absent. In case of a positive HPV-detection we recommended to have a timely cytology performed, as is recommended in the German national guideline for prevention of cervical cancer (13). Male PID patients with visible pathologies like papulae and positivity for HPV-DNA in genital swabs were advised to consult a dermatologist to remove the papulae.

Only two females of our PID patients were vaccinated against HPV, i.e. a HPV-vaccination rate of 7% in the whole study population and 13% among female patients. Not only low acceptance of the HPV-vaccination, as described in the general population (35), might be a reason for the low vaccination status, but also the age of our cohort with a median age of 38.9 years. Only the two described females were eligible to receive a HPV-vaccination according to their age. Our patients were thus mostly too old to receive HPV-vaccination within the recommendation of the German national vaccination guidelines, as the German recommendations for HPV-vaccination were published in 2007 for girls and in 2018 for boys from 9 to 14 years of age (8, 9).

We found differences in the lymphocyte subsets between HPV-positive and HPV-negative PID patients. The investigation of T cells in PID patients is important to detect combined T and B cell defects and to identify patients with low naïve T helper cells, who are at risk of opportunistic infections and more often need antibiotics despite immunoglobulin replacement therapy (36). HPV-positive patients had higher T cells, higher transitional B cells and higher circulating plasmablasts. This correlation of high T cell numbers might be unexpected, as usually low T cell counts are associated with viral infections. The higher T cell numbers might be explained by a more lymphoproliferative phenotype of the PID with increased lymphocyte numbers in HPV-positive patients. Another explanation might be a stimulation of the lymphocytes by chronic viral infection. The significance of higher transitional B cells and plasmablasts is unclear. These findings in the B cell compartment might be influenced by the former rituximab treatment, which three of our patients had received. Due to the small sample size, the correlations we describe between HPV-status and lymphocyte subsets should be evaluated in a bigger cohort, to allow performance of multivariate analysis and to detect confounding factors on lymphocyte numbers.

What limits our study, is the lack of genetic analysis for PID diagnosis. Our patients were diagnosed using clinical criteria. Additionally, in future studies, anal and oral swabs should be included to detect HPV in additional locations and a longitudinal follow-up should be performed to answer if and when clearing of genital HPV takes place and if asymptomatic HPV-infection is associated with an increased rate of the occurrence of HPV-associated malignancies. Furthermore, to identify risk factors for HPV-infection and -malignancy, a larger cohort of PID patients should be tested.

## Conclusion

Patients with PIDs show a high rate of HPV-positivity in genital swabs with a high rate of high-risk HPV-types in our cohort. As PID patients are susceptible to malignancy, regular screenings for HPV-positivity and consecutively for HPV-associated malignancy should be performed. Particularly high-risk HPV-infections should receive stringent follow-ups. To reduce the HPV-prevalence in PID patients the HPV-vaccination rate should be increased.

## Data Availability Statement

The raw data supporting the conclusions of this article will be made available by the authors, without undue reservation.

## Ethics Statement

The studies involving human participants were reviewed and approved by ethics committee of the University of Würzburg. The patients/participants provided their written informed consent to participate in this study.

## Author Contributions

MG had full access to all of the data in the study and takes responsibility for the integrity of the data and the accuracy of the data analysis. Study conception and design: MG and ES. Acquisition of data: MG, MK, MF, P-PS, JP, MS, and H-PT. Analysis and interpretation of data: MG, ES, RR, H-PT, and MS. All authors were involved in drafting the article or revising it critically for important intellectual content, and all authors approved the final version to be submitted for publication.

## Funding

This publication was supported by the Open Access Publication Fund of the University of Würzburg.

## Conflict of Interest

MG received travel grants from AbbVie, Chugai, Eli Lilly, Hexal, Janssen, Novartis, Pfizer and compensation for board membership from Takeda. MF received travel grants from AbbVie, Novartis, Janssen, Eli Lilly and compensation for board memberships from AbbVie. P-PS received travel grants from AbbVie, Eli Lilly, Janssen-Cilag. JP received travel grants from AbbVie and Janssen-Cilag. H-PT received speaker’s fees, travel grants, research funding, or compensation for consultancies or board memberships from AbbVie, Chugai/Roche, Eli Lilly, Gilead, Janssen, Novartis, Sandoz/Hexal, Sanofi Aventis, Takeda (Shire). MS received speaker’s fees, travel grants, research funding, or compensation for consultancies or board memberships from AbbVie, Actelion, BMS, Boehringer/Ingelheim, Celgene, Chugai/Roche, Eli Lilly, Genzyme, Gilead, Hexal/Sandoz, Janssen-Cilag, MSD, Novartis, Pfizer, Sanofi Pasteur, Takeda (Shire), UCB. ES received speaker’s fees, travel grants, research funding, or compensation for consultancies or board memberships from AbbVie, Chugai/Roche, Janssen-Cilag, Eli Lilly, Novartis, Pfizer, Takeda (Shire).

The remaining authors declare that the research was conducted in the absence of any commercial or financial relationships that could be construed as a potential conflict of interest.

## Publisher’s Note

All claims expressed in this article are solely those of the authors and do not necessarily represent those of their affiliated organizations, or those of the publisher, the editors and the reviewers. Any product that may be evaluated in this article, or claim that may be made by its manufacturer, is not guaranteed or endorsed by the publisher.

## Abbreviations

APC, allophycocyanin; CD, cluster of differentiation; CID, combined immunodeficiency; CVID, common variable immunodeficiency; ECD, phycoerythrin-Texas Red; EDTA, ethylenediaminetetraacetic acid; EMA, European medicines agency; ESID, European society for immunodeficiencies; FACS, fluorescence-activated cell sorting; FITC, IgD-fluorescein isothiocyanate; HIV, human immunodeficiency virus; HPV, human papillomavirus; Ig, immunoglobulin; IQR, interquartile range; IARC, International agency for research on cancer; na, not applicable; NK, natural killer; PB, Pacific Blue; PC, phycoerythrin-cyanin; PE, phycoerythrin; PID, primary immunodeficiency; SLE, systemic lupus erythematosus.
